# Glycyrrhizin as antiviral agent against Hepatitis C Virus

**DOI:** 10.1186/1479-5876-9-112

**Published:** 2011-07-18

**Authors:** Usman A Ashfaq, Muhammad S Masoud, Zafar Nawaz, Sheikh Riazuddin

**Affiliations:** 1 Division of Molecular Medicine, National Centre of Excellence in Molecular Biology, University of the Punjab, Lahore, Pakistan; 2 Braman Family Breast Cancer Institute, University of Miami, USA; 3 Allama Iqbal Medical College, University of Health sciences, Lahore

## Abstract

**Background:**

Hepatitis C virus is a major cause of chronic liver diseases which can lead to permanent liver damage, hepatocellular carcinoma and death. The presently available treatment with interferon plus ribavirin, has limited benefits due to adverse side effects such as anemia, depression, fatigue, and "flu-like" symptoms. Herbal plants have been used for centuries against different diseases including viral diseases and have become a major source of new compounds to treat bacterial and viral diseases.

**Material:**

The present study was design to study the antiviral effect of Glycyrrhizin (GL) against HCV. For this purpose, HCV infected liver cells were treated with GL at non toxic doses and HCV titer was measured by Quantitative real time RT-PCR.

**Results and Discussion:**

Our results demonstrated that GL inhibit HCV titer in a dose dependent manner and resulted in 50% reduction of HCV at a concentration of 14 ± 2 μg. Comparative studies were made with interferon alpha to investigate synergistic effects, if any, between antiviral compound and interferon alpha 2a. Our data showed that GL exhibited synergistic effect when combined with interferon. Moreover, these results were verified by transiently transfecting the liver cells with HCV 3a core plasmid. The results proved that GL dose dependently inhibit the expression of HCV 3a core gene both at mRNA and protein levels while the GAPDH remained constant.

**Conclusion:**

Our results suggest that GL inhibit HCV full length viral particles and HCV core gene expression or function in a dose dependent manner and had synergistic effect with interferon. In future, GL along with interferon will be better option to treat HCV infection.

## Background

Hepatitis C virus (HCV) is a major cause of liver associated diseases all over the world. An estimated 3% of the world's populations, (more than 350 million people) are chronically infected by HCV, which is the main cause of liver fibrosis, cirrhosis and hepatocellular carcinoma (HCC) [1]. Like other RNA viruses, HCV possess a high degree of sequence variability that likely contributes to its ability to establish chronic infections after a mild acute phase. Current treatment of standard for HCV comprises a combination of high-dose pegylated interferon alpha (IFN-α) with the guanosine analogue ribavirin (Rib). About 75% of patients receive no therapeutic benefit from the current combination therapy with PEG-IFN α and the guanosine analog ribavirin because of adverse side effects and high cost [2]. Vaccine development is hindered by the lack of good *in-vitro *and *in-vivo *models of infection, the antigenic heterogeneity of the virus and its ability to avoid immune defenses. Hence, there is a need to develop antiviral drug to treat Hepatitis infection from plant sources.

The HCV is an enveloped positive-stranded RNA virus belonging to the *Hepacivirus *genus of the Flaviviridae family. HCV has six major genotypes and approximately 100 subtypes depending on the geographical distribution of the virus [3]. HCV genome encodes a single polyprotein precursor of approximately 3000 amino acid residues replicated in the cytosol through a negative-strand intermediate. An internal ribosome entry site (IRES) drives translation of the polyprotein, which is co- and post-translationally processed by cellular and viral proteases to yield mature viral structural proteins Core, E1 and E2, and nonstructural proteins NS2, NS3, NS4A, NS4B, NS5A and NS5B, while an additional protein can be produced by a ribosomal frameshift in the N-terminal region of the polyprotein [4,5]. HCV structural proteins (core, E1 and E2) and nonstructural proteins (NS3 protease and NS5B RNA-dependent RNA polymerase) are potent molecular targets of new antiviral compounds.

Glycyrrhiza glabra is a perennial herb, native to central and South-Western Asia, as well as to the Mediterranean region and is cultivated in temperate and sub-tropical regions of the world, including Europe and Asia. Dried roots of Glycyrrhiza glabra have a characteristic odour and sweet taste. It has anti-inflammatory, antioxidant and immunomodulatory activities. Glycyrrhizin is the major component of Glycyrrhiza glabra root, at concentrations of 1-9%. Glycyrrhizin is a glycosylated saponin, containing one molecule of glycyrretinic acid, with structural similarities to hydrocortisone, and two molecules of glucuronic acid [6,7]. It has been attributed to numerous pharmacologic effects like anti-inflammatory, anti-viral, anti-tumor, and hepatoprotective activities [8]. It has been shown that GL inhibited the inflammation in mice model of liver injury [9].

The present study was undertaken to study the effect of GL against HCV 3a in liver cells. We report here that GL effectively inhibited HCV full length viral particles and HCV 3a Core gene RNA and protein expression in a dose-dependent manner in Huh-7 cells.

## Material and Methods

### Serum Sample Collection

The local HCV-3a patient's serum samples used in this investigation were obtained from the CAMB (Center for Applied Molecular Biology) diagnostic laboratory, Lahore, Pakistan. Serum samples were stored at -80°C prior to viral inoculation experiments. Quantification and genotype was assessed by CAMB diagnostic laboratory, Lahore, Pakistan. Patient's written consent and approval for this study was obtained from institutional ethics committee.

### Cell line

The Huh-7 cell line was offered by Dr. Zafar Nawaz (Biochemistry and Molecular Biology Department, University of Miami, USA). Huh-7 cells were cultured in Dulbecco's modified Eagle medium (DMEM) supplemented with 10% fetal bovine serum & 100 IU/ml penicillin & 100 μg/ml streptomycin, at 37°C in an atmosphere of 5% CO_2_.

### Plasmid construction

For the construction of expression plasmid, viral RNA was isolated from 100 μl serum aliquots using Gentra RNA isolation kit (Gentra System Pennsylvania, USA) according to the manufacturer's instructions. 100-200 ng extracted viral RNA was used for RT-PCR using the SuperScript III one-step RT-PCR system (Invitrogen Life technologies, USA). HCV complementary DNA (cDNA) encoding the full length Core protein (amino acid 1-191 of HCV-3a) were amplified and cloned into pCR3.1 mammalian expression plasmid (kindly provided by Dr. Zafar Nawaz, University of Miami, USA) with Flag TAG inserted at the 5' end of the Core gene with EcoRV and XbaI restriction sites.

### Cellular toxicity through Trypan blue dye explosive method

Trypan blue dye was used for confirmation of viability of Huh-7 and CHO cells. For toxicological analysis of GL, liver cells were seeded at a density of 3 × 10^5 ^in six well plate. First well was considered as control and added different concentrations of the GL from lowest to highest in the remaining wells. After 24 h trypsinized the cells, prepared a suspension of 1:1 of the cell suspension to trypan blue dye and dispensed 10 μl of it on a glass slide and counted viable cells through haemocytometer.

### Anti-HCV analysis of Glycyrrhizin on Huh-7 cells

Huh-7 cell line was used to establish the in-vitro replication of HCV. A similar protocol was used for viral inoculation as established by Zekari et al. 2009 [10] and El-Awardy et al. 2006 [11]. High viral titer > 1 × 10^8 ^IU/ml from HCV-3a patient's was used as principle inoculum in these experiments. Huh-7 cells were maintained in 6-well culture plates to semi-confluence, washed twice with serum-free medium, then inoculated with 500 μl (5 × 10^7^IU/well) and 500 μl serum free media. Cells were maintained overnight at 37°C in 5% CO_2_. Next day, adherent cells were washed three times with 1× PBS, complete medium was added and incubation was continued for 48 hrs. Cells were harvested and assessed for viral RNA quantification by Real Time PCR. To analyze the effect of GL on HCV infection, serum infected Huh-7 cells were again seeded after three days of infection in 24-well plates in the presence and absence of GL and grown to 80% confluence with 2 ml medium. After 24 h, cells and total RNA was isolated by using Gentra RNA isolation kit (Gentra System Pennsylvania, USA) according to the manufacturer's instructions. Briefely, cells were lysed with cell lysis solution containing 5 μl internal control (Sacace Biotechnologies Caserta, Italy). RNA pallet was solubilized in 1% DEPC (Diethyl pyrocarbonate treated water). HCV RNA quantifications were determined by Real Time PCR Smart Cycler II system (Cepheid Sunnyvale, USA) using the Sacace HCV quantitative analysis kit (Sacace Biotechnologies Caserta, Italy) according to the manufacturer's instructions.

### Formula for the calculation of HCV RNA concentration

Following formula was used to calculate the concentration HCV RNA of each sample.

IC = internal control, which is specific for each lot.

### Antiviral activity of GL against HCV 3a core gene

For transfection studies, Huh-7 cells (5 × 10^4^) were plated in 24-well plates for 24 h. The medium was removed and cells were washed with 1× PBS. Cells were transiently transfected with expression plasmids containing HCV 3a core gene (0.4 μg) in the presence and absence of GL by using Lipofectamine™ 2000 (Invitrogen life technologies, Carlsbad, CA) according to the manufacturer's protocol. Total RNA was extracted by using Trizol reagent (Invitrogen life technologies, Carlsbad, CA) according to the manufacturer's protocol. To analyze the effect of GL against HCV 3a core gene, cDNA was synthesized with 1 μg of RNA, using Revert Aid TM First Strand cDNA Synthesis Kit (Fermentas, St. Leon-Rot/Germany). Gene expression analysis was carried out via PCR (Applied Biosystems Inc, USA) by using 2X PCR Mix (Fermentas). Following primers were used for the amplification of HCV Core forward primer: GGACGACGATGACAAGGACT; HCV core reverse: GGCTGTGACCGTTCAGAAGT; GAPDH Forward: ACCACAGTCCATGCCATCAC: and GAPDH reverse; TCCACCACCCTGTTGCTGTA PCR was performed by initial denaturation at 95°C for 5 min followed by 30 cycles, each of denaturation at 92°C for 45s, annealing at 58°C for 45 s, and extension at 72°C for 1 min, with final extension at 72°C for 10 min. The amplified DNA samples were analyzed on 2% agarose gel. The DNA bands were visualized directly under the UV and the photographs of the gels were obtained with gel documentation system.

### Western Blotting

To determine the protein expression levels of HCV Core, the transfected and non-transfected cells were lysed with ProteoJET mammalian cell lysis reagent (Fermentas, Canada). Equal amounts of total protein were subjected to electrophoresis on 12% SDS-PAGE and electrophoretically transferred to a nitrocellulose membrane following the manufacturer's protocol (Bio-Rad, CA). After blocking non-specific binding sites with 5% skimmed milk, blots were incubated with primary monoclonal antibodies specific to HCV Core and GAPDH (Santa Cruz Biotechnology Inc, USA) and secondary Horseradish peroxidase-conjugated anti-goat anti-mouse antibody (Sigma Aldrich, USA). The protein expressions were evaluated using chemiluminescence's detection kit (Sigma Aldrich, USA).

## Results

### Toxicological study of GL in liver and fibroblast cells

**Figure 1:**
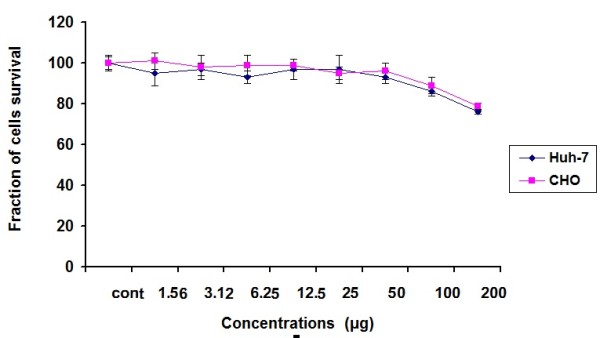
**Toxicological study of GL in Huh-7 and CHO cell**: Huh-7 and CHO cells were plated at the density of 4 × 10^4 ^in six well plates. After 24 h cells were treated with different concentrations of GL and control consisted of solvent in which compound is disolved. After 24 h incubation period cells were trypsinized and counted with haemocytometer and trypan blue dye explosive method.

Cytotoxic effects of GL was analyzed after 24 h incubation of Huh-7 and CHO cells with the concentration of 3.125, 6.25, 12.5, 25, 50 and 100 μg/ml. Cell viability was evaluated using a viability dye and counting the cells through haemocytometer. Figure 1 shows cytotoxicity analysis of GL and demonstrates that Huh7 and CHO cells viability is unaffected up to a concentration of 100 μg. However, when exceeds from 100 μg, toxic effect in liver and fibroblast cells were observed. The data verified by microscopic examination of cells and MTT cell proliferation assay demonstrate that GL has no toxic effect at 100 μg concentration (data not shown).

### Antiviral effect of GL against HCV

**Figure 2:**
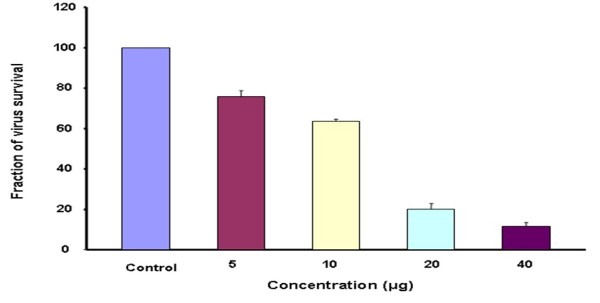
**Dose dependent inhibition of GL against HCV 3a genotype**. Huh 7 cells were infected with 2 × 10^5 ^copies of HCV 3a genotype per well in the absence and presence of different concentrations of GL. After 24 h incubation period, total RNA was extracted by Gentra kit, and the levels of HCV RNA remaining were determined by real time Quantitative RT-PCR assay and are shown as percentage of HCV RNA survival in cells. P value > 0.05 vs control was considered as statistically significant.

To determine the antiviral effect of GL, Huh-7 cells were plated at the density of 3 × 10^5 ^cells in six well plates. After 24 h, cells were infected with 2 × 10^5 ^HCV virus copies of 3a genotype in the presence and absence of different concentrations of GL. Cells were incubated at 37°C in CO_2 _incubator for additional 24 h. At the end of the incubation, cells were lysed with cell lysis solution. Total RNA was extracted through Gentra RNA isolation kit and HCV titer was determined with real time RT PCR through HCV specific labeled primer. The results of our study demonstrate that GL has antiviral effect against HCV in a dose-dependent manner (Figure 2). Real time RT-PCR results exhibited that GL resulted in 50% reduction of HCV at a concentration of 14 ± 2 μg. At a 40 μg concentration, viral inhibition of GL reached up to 89%.

### Synergistic effect of GL along with interferon

**Figure 3:**
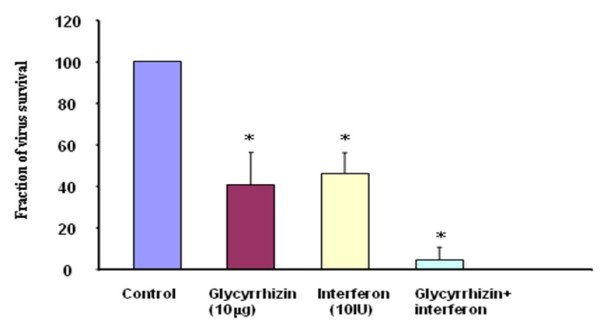
**Synergy in the antiviral activity of GL along with interferon**. GL shows synergistic effect with interferon-α (5 IU/well) against HCV in liver cells (Huh-7). Huh-7 cells were incubated for 6 h with GL and interferon alone, or combination of GL and interferon in a 96-well plate. After 6 h cells were infected with 2 × 10^4 ^copies of HCV 3a genotype per well and incubated for additional 18 h. At the end of incubation period, total RNA was extracted by Gentra kit, and the levels of HCV RNA remaining were determined, by real time Quantitative RT-PCR assay and are shown as percentage of HCV RNA survival in cells. Results are represented as the average and standard error for three independent experiments. *P value > 0.05 vs control.

After the dose response analysis, the synergistic effect of GL was checked along with interferon. Cells were seeded at 2 × 10^4 ^cells per well in 96-well plates in DMEM medium supplemented with 10% FBS and pre-incubated for 24 h. Cells were then treated with 10 IU IFN-alpha 2b for 6 h and were incubated with HCV 3a for additional 18 h. The effect of the compound was tested with or without interferon and viral titers were quantified through Quantitative RT-PCR. Figure 3 shows that GL exhibited 55% reduction in viral titer alone but when GL was combined with interferon, it resulted in 95% reduction in viral titer.

### Antiviral effect of GL against HCV Core gene

**Figure 4:**
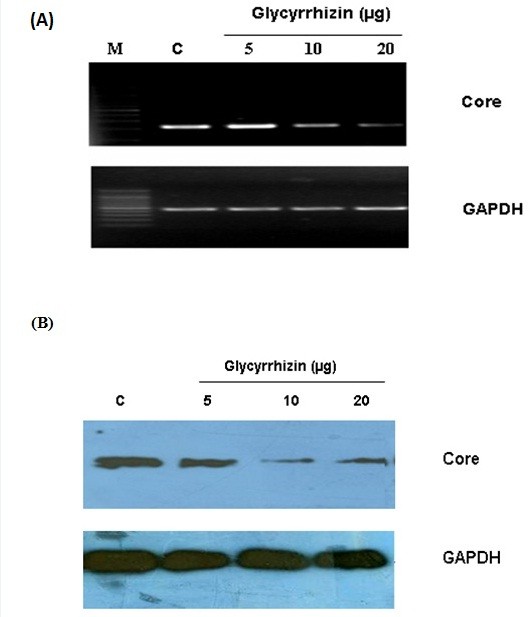
**Dose dependent inhibition of GL against HCV core gene**. Huh-7 cells were transfected with Core in the presence and absence of different concentration of GL. (A) After 24 h incubation period, total RNA was extracted and the levels of HCV core gene were determined by RT-PCR. GAPDH serve as internal control. (B) After 48 h incubation period, protein were isolated and analyzed by western blotting with anti -Core monoclonal antibody and GAPDH served as internal control.

To determine the antiviral effect against HCV core gene, Huh-7 cells were transfected with HCV core gene in the presence and absence of different concentrations of GL. After 24 h, RNA was extracted through Triazol (Invitrogen). cDNA were generated by oligo dT primer. cDNA was amplified by PCR using primers specific to the HCV core gene of 3a genotype. Amplification of GAPDH mRNA served as an internal control. Figure 4 demonstrates that GL inhibits HCV RNA and protein expression significantly in a dose-dependent manner, while GAPDH mRNA and protein expression remains unaffected by the addition of the GL.

## Discussion

HCV infection is a serious global health problem necessitating effective treatment. Currently, there is no vaccine available for prevention of HCV infection due to high degree of strain variation. The current treatment of care, Pegylated interferon α in combination with ribavirin is costly, has significant side effects and fails to cure about half of all infections [12,13]. Hence, there is a need to develop anti-HCV agents, both from herbal and synthetic chemistry, which are less toxic, more efficacious and cost-effective. Previous studies demonstrated that medicinal plants used for centuries against different diseases including viral diseases and become a focal point to identify, isolate and purify of new compounds to treat diseases such as Hepatitis. Many traditional medicinal plants and herbs were reported to have strong antiviral activity against DNA and RNA viruses by inhibiting virus replication, interfering with virus-to-cell binding and immunomodulation action [14,15]. HCV structural proteins (core, E1 and E2) and nonstructural proteins (NS3 protease and NS5B RNA-dependent RNA polymerase) are potent molecular targets of new antiviral compounds.

GL (licorice root extract) has anti-inflammatory and antioxidant activities. GL inhibits CD4+ T-cell and tumor necrosis factor (TNF)-mediated cytotoxicity [16]. GL has a membrane stabilizing effect [17] and also stimulates endogenous production of interferon [18]. 18-β glycyrrhetinic acid, an active constituent of Glycyrrhizic acid shows antiviral activity against a number of DNA and RNA viruses possibly due to activation of NFκB and induction of IL-8 secretion [19]. GL has been used in Japan for more than 20 years orally and as the intravenous drug Stronger Neo-Minophagen C (SNMC). Oral GL is metabolized in the intestine to a compound called glycyrrhetinic acid (GA) and intravenous GL is metabolized into glycyrrhetinic acid when excreted through the bile into the intestines. GL and glycyrrhetinic acid have both been tested against Hepatitis A, B, C--with some interesting results [20-22]. Previous studies report that GL has antiviral activity against HIV by inhibiting virus replication, interfering with virus-to-cell binding and cell-to-cell infection, and inducing IFN activity [23,24]. GL has reported antiviral effect against Herpesviridae family viruses (VZV, HSV-1, EBS, CMV) and Flaviviruses by inhibiting the replication of virus [7,25]. GL has also antiviral effect against some emerging viruses such as SARS by inhibiting the virus replication and production of NO synthase [26] The results of our study show that GL has antiviral effect against HCV at non toxic concentrations. Firstly, GL was checked for toxicological analysis in both Huh-7 and CHO cell lines. Our data shows that GL is non toxic at concentrations up to 100 μg (Figure 1). The data was further verified by microscopic examination of cells and MTT cell proliferation assay [27].

Guha et al. [28] reported that in vitro cell culture models can at best demonstrate the infectivity of the virus and used in evaluating drugs for antiviral activity or inhibition of HCV infection. Most of the studies all over the world are conducted in Huh-7 derived cell lines and with replicons supporting HCV RNA transcription and protein synthesis. Recently different groups have studied the HCV replication in serum infected liver cell lines for the study of different HCV genotypes which mimics the naturally occurring HCV virions biology and kinetics of HCV infection in humans [29,30]. We infected Huh-7 cells with native viral particles from HCV 3a positive serum, the most prevalent type in Pakistan using the same protocol as established [29]. The results of our data demonstrate that GL has antiviral effect against HCV in a dose-dependent manner (Figure 2). The results prove that GL showed 50% reduction of HCV at a concentration of 13 μg. At a concentration of 40 μg, viral inhibition by the GL reached up to 85%.

HCV Core protein modulates gene transcription, cell proliferation, cell death and cell signaling, interferes with metabolic genes and suppresses host immune response [31] leading to oxidative stress, liver steatosis and eventually hepatocellular carcinoma [32]. Core protein is also able to up-regulate cyclooxygenase-2 (Cox-2) expression in hepatocytes derived cells, providing a potential mechanism for oxidative stress [33]. The expression of Cox-2 in HCC was found to correlate with the levels of several key molecules implicated in carcinogenesis such as inducible nitric oxide synthetase (iNOS), activate vascular endothelial growth factor (VEGF) and phosphorylated Akt (p-Akt) [34,35]. Our data shows that GL inhibits HCV core gene expression or function in a dose-dependent manner similar to interferon alpha 2a. This may be due to stimulation of interferon pathway by phosphorylation of Stat1 on tyrosine and serine [36]. GL may show antiviral effect due to its ability to reduce membrane fluidity [37] and up regulation of Cox2 or related pathway.

## Conclusion

GL inhibits HCV full length viral particle and HCV core gene expression both at RNA and protein level and had synergistic effect with interferon. Therefore, it can also be speculated from our pilot study that therapeutic induction of GL either alone or in combination with IFN treatment might represent an alternative approach for future treatment of chronic infection.

## Abbreviations

**HCV**: Hepatitis C virus; **GL: **Glycyrrhizin; **Huh-7**: Human Hepatoma Cell line.

## Competing interests

The authors declare that they have no competing interests.

## Authors' contributions

UAA contributed in lab work and manuscript writes up. MSM helped me in cell culture. SRD and ZN was the principal investigator and provide all facilitates to complete this work. All the authors read and approved the final manuscript.

## Authors' information

Usman Ali Ashfaq (PhD Molecular Biology), Sheikh Riazuddin (PhD molecular Biology and Dean Post graduate study at Allama Iqbal medical college, Lahore
